# Development and validation of a ferroptosis-related lncRNAs prognosis signature in colon cancer

**DOI:** 10.17305/bjbms.2020.5617

**Published:** 2021-10

**Authors:** Hua-jun Cai, Zhi-cheng Zhuang, Yong Wu, Yi-yi Zhang, Xing Liu, Jin-fu Zhuang, Yuan-feng Yang, Yuan Gao, Bin Chen, Guo-xian Guan

**Affiliations:** Department of Colorectal Surgery, The First Affiliated Hospital of Fujian Medical University, Fuzhou, China

**Keywords:** TCGA, colon adenocarcinoma, ferroptosis, long non-coding RNA, prognosis signature

## Abstract

Ferroptosis is a form of iron-dependent programmed cell death. Regulate ferroptosis in tumor cells is a novel treatment modality. The present study aimed to investigate ferroptosis-related long non-coding RNAs (lncRNAs) and construct a prognostic model for colon adenocarcinoma (COAD). RNA- sequencing data and ferroptosis-related genes were obtained from The Cancer Genome Atlas database and FerrDb database. COAD patients were randomly assigned to training- and validation groups. The Least Absolute Shrinkage and Selection Operator regression and Cox regression model were used to determine and develop a predictive model. The model was corroborated using the validation group and the entire group. In total, 259 ferroptosis-related genes and 905 ferroptosis-related LncRNAs were obtained. Cox model revealed and constructed seven ferroptosis-related LncRNAs signature (LINC01503, AC004687.1, AC010973.2, AP001189.3, ARRDC1-AS1, OIP5-AS1, and NCK1-DT). Patients were assigned into two groups according to the median risk score. Kaplan–Meier survival curves showed that overall survival between high- and low-risk groups was statistically significant (P<0.01). Cox multivariate analysis seven ferroptosis-related LncRNAs signature was an independent risk factor for COAD outcomes (P<0.05). The relationship between seven ferroptosis-related LncRNAs and clinicopathological features was also examined. The principal component analysis showed a difference between high- and low-risk groups intuitively. With the aid of gene set enrichment analysis, the underlying mechanisms of seven ferroptosis-related LncRNAs were uncovered, including the MAPK signaling pathway, mTOR signaling pathway, and glutathione metabolism pathway. Finally, we established and validated seven ferroptosis-related lncRNAs signature for COAD patients to predict survival. These results may provide meaningful targets for future study.

## INTRODUCTION

Colon adenocarcinoma (COAD) is a common malignant tumor worldwide, which endangers human health[1]. Along with advances in medicine, personalized cancer therapy is an emerging trend. Thus, more investigation into useful prognostic biomarkers and the possible therapeutic target is required.

Ferroptosis is a form of iron-dependent programmed cell death, characterized by lipid peroxidation[2]. With the deepening of research, ferroptosis is involved in diverse vital biological processes, including cancer, ischemia-reperfusion injury, and neurodegenerative diseases[3, 4]. Regulate ferroptosis in tumor cells is a novel treatment modality[5]. Increasing evidence supports that ferroptosis participates in the pathophysiology of COAD[6-8]. Thus, identifying critical regulators of ferroptosis is an essential step towards a more in-depth understanding.

Long non-coding RNA (lncRNA) refers to a type of non-coding RNAs with more than 200 nucleotides in length. By mediating chromosome modification, transcriptional activation, and interference, lncRNA regulates diverse physiological and biochemical cellular processes[9, 10]. The LINC00336 was found to inhibit lung cancer ferroptosis by interacting with ELAVL1[11]. The lncRNA MT1DP increases non-small cell lung cancer sensitivity to ferroptosis through regulate miR-365a-3p/NRF2 axis[12]. Recent studies demonstrated that the lncRNA GABPB1-AS1 downregulated the PRDX5 peroxidase gene expression and reduced liver cellular antioxidant capacity [13]. Furthermore, high GABPB1 expression in hepatocellular carcinoma was associated with a poor prognosis. Accordingly, there is a reason to identify ferroptosis-related lncRNAs to predict COAD patients’ prognosis, which can provide a foundation for individualized treatment.

In the present study, we obtained COAD RNA sequencing (RNA-seq) data in 417 patients downloaded from The Cancer Genome Atlas (TCGA) database and assigned patients to training and validation datasets. Basing on the training dataset, we identified seven ferroptosis-related lncRNA with prognostic value and developed a lncRNA signature prognostic model, which might represent potential therapeutic targets. Finally, we verified the predictive capacity of the model in the validation dataset and the entire dataset.

## MATERIALS AND METHODS

### Expression data acquisition

RNA-seq FPKM (reads per kilobase per million) and clinical data were obtained from the cancer genome atlas (TCGA) website (https://portal.gdc.cancer.gov/). Annotation of lncRNAs was obtained from the GENCODE website (https://www.gencodegenes.org/). Clinical data including age, sex, pT stage, pN stage, pM stage, AJCC stage, and follow-up data were collected. Patients with incomplete survival data were eliminated. Considering non-cancer-related deaths, we also excluded patients with survival time <30 days[14]. Finally, a total of 417 patients were included. Subsequently, with the R package “caret”, cases were randomly assigned to the training group (n = 209) and validation group (n=208).

### Identification of ferroptosis-related lncRNAs

We downloaded the ferroptosis-related genes set from FerrDb, the first manually curated resource for regulators and markers of ferroptosis and ferroptosis-disease associations, released in January 2020 (http://www.zhounan.org/ferrdb) [15]. We performed the “limma” package to calculate the correlation of expression between ferroptosis-related genes and lncRNAs and identified ferroptosis-related lncRNAs. The threshold was set as the correlation coefficient > 0.4 and P-value < 0.001.

### Prognosis lncRNAs signature development

We evaluated each lncRNA’s prognostic value by univariate Cox regression analysis (p < 0.05), and after filtering, 56 prognostic ferroptosis-related lncRNA remained. Further screening was based on the Least Absolute Shrinkage and Selection Operator (LASSO) regression to avoid overfitting. Next, to determine final prognostic ferroptosis-related lncRNA, the multivariate Cox regression analysis was used. We then constructed a lncRNAs signature with the lowest Akaike information criteria (AIC) value. The risk score of each COAD patients was calculated on the basis of the amount of lncRNA expression and corresponding coefficient. Specifically, risk score =β_lncRNA_1×Expression_lncRNA_1+β_lncRNA_2×Expression_lncRNA_2+β_lncRNA_3×Expression_lncRNA_3+…+ β_lncRNA_n×Expression_lncRNA_n.

### Prediction capacity of risk score model

Using the median risk score value and the corresponding coefficient of the training group, patients in training- and validation groups were divided into high- and low-risk groups. Survival curves were generated using the Kaplan-Meier method. The receiver operating characteristic (ROC) curve and the area under the ROC curve (AUC) were performed to evaluated predictive power. The validation group and the entire cohort were applied to validate this model. Univariate and multivariate Cox regression analyses were conducted to assess the prognostic value of the risk score model. What’s more, univariate analysis was performed for each clinicopathological features.

### PCA and GSEA

Principal component analysis (PCA) was conducted to reduce dimension and visualize different ferroptosis statuses base on high- and low-risk groups. Besides, potential biological functions were investigated by gene set enrichment analysis (GSEA 4.1.0), and P < 0.05 and FDR < 0.25 were regarded as statistically significant.

### Statistical analysis

The statistical analyses were performed using the R (www.r-project.org). Clinicopathological characteristics were compared within the training- and validation groups using the Chi-square test. The Pearson correlation test was used to analyze correlations. Statistical significance was set at P<0.05.

## RESULTS

### Data acquisition

The clinicopathological characteristics of 417 COAD patients were detailed in Table 1. The training group and validation group was comparable. In total, 259 ferroptosis-related genes were collected from FerrDb (Appendix 1). Using Pearson’s correlation test, 905 ferroptosis-related lncRNAs were obtained.

**TABLE 1:**
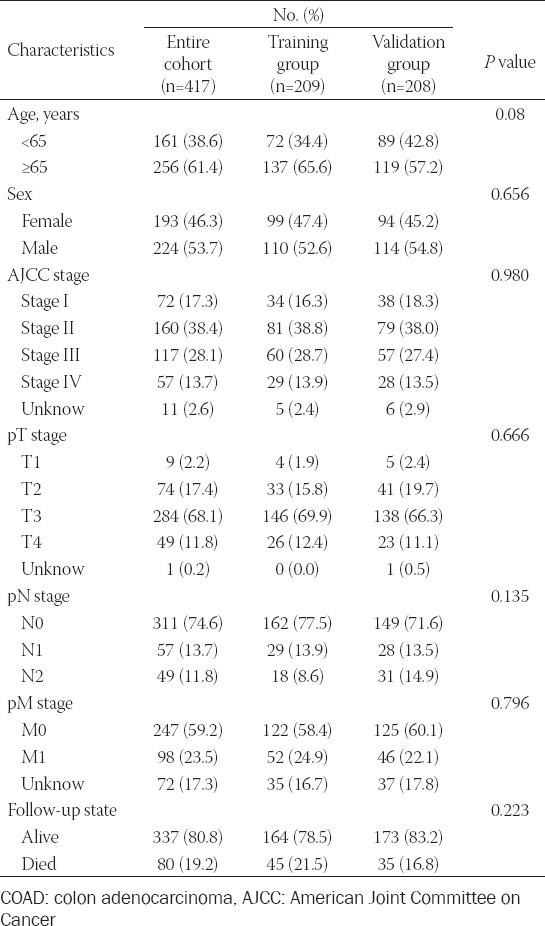
Characteristics of COAD patients

### Development and validation of prognosis lncRNAs signature

**FIGURE 1:**
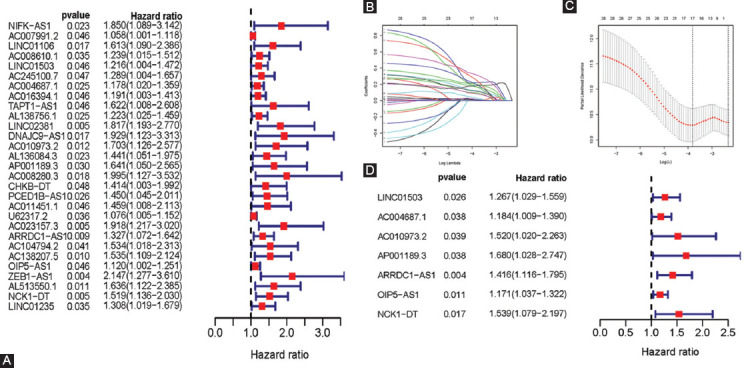
Construction of ferroptosis-related lncRNAs prognostic signature. (A) Univariate Cox regression illustrated twenty-seven ferroptosis-related lncRNAs associated with prognosis. (B, C) Seventeen ferroptosis-related lncRNAs were identified by LASSO regression analysis. (D) Multivariate Cox regression analysis revealed seven ferroptosis-related lncRNAs were independent prognostic factors for COAD patients.

**FIGURE 2:**
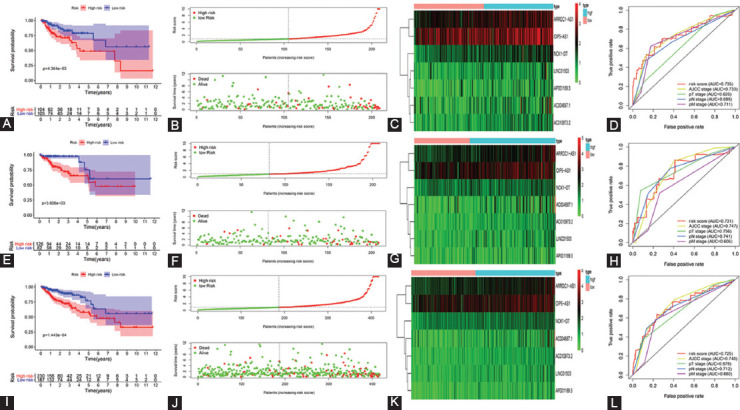
Validation of ferroptosis-related lncRNAs prognostic signature. (A) KM curve, (B) risk score, (C) heatmap, and (D) ROC curve of the prognostic signature in the training group. (E) KM curve, (F) risk score, (G) heatmap, and (H) ROC curve of the prognostic signature in the validation group. (I) KM curve, (J) risk score, (K) heatmap, and (L) ROC curve of the prognostic signature in the entire group.KM curve: Kaplan-Meier curve, ROC curve: receiver operating characteristics curve.

We constructed lncRNAs signature based on the training group. For preliminary screening, univariate Cox analysis showed 29 ferroptosis-related lncRNAs associated with overall survival (OS, Figure 1A-1B). Next, 17 lncRNAs were selected by Lasso regression (Figure 1C-1D). Finally, a multivariate Cox regression analysis revealed seven ferroptosis-related lncRNAs were independent prognostic factors for COAD patients. To this end, we generated seven ferroptosis-related lncRNAs signature to predict prognosis. Specifically, for each patient, the risk score was calculated according to the followed equation. Risk score: 0.236 × Expression_LINC01503_ + 0.169 × Expression_AC004687.1_ + 0.418 × Expression_AC010973.2_ + 0.519 × Expression_AP001189.3_ + 0.347 × Expression_ARRDC1-AS1_ + 0.158 × Expression_OIP5-AS1_ + 0.431 × Expression_NCK1-DT_. Based on the median risk score, patients were divided into a high- and low-risk group. KM curves demonstrated high-risk group has a worse prognosis (Figure 2A). The distribution patterns of risk scores and survival status were observed (Figure 2B). A heatmap exhibited expressed of seven ferroptosis-related lncRNAs between the high- and low-risk group (Figure 2C). Then, we performed a ROC curve to assess the accuracy of the risk score model. AUC value was 0.735 (Figure 2D). For the validation group and the entire group, we also observed differences in OS between high- and low-risk groups were of statistical significance (all P < 0.05; Figure 2E-F,2I-J). Figure 2G and 2K demonstrate the expression of seven ferroptosis-related lncRNAs between the high- and low-risk group in the validation group and the entire group, respectively. AUC values were 0.731 for the validation group and 0.725 for the entire group (Figure 2H,2L), which verified the lncRNAs signature’s accuracy.

### Independent prognostic factors of OS

**FIGURE 3:**
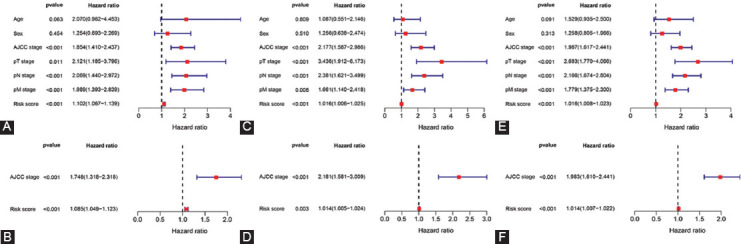
The independent prognostic factors for COAD OS. (A, C, E) Univariate Cox regression of prognostic factors in the training-, validation- and entire group, respectively. There were statistically significant differences among AJCC stage, T stage, N stage, M stage, and risk score. (B, D, F) Multivariate cox regression of prognostic factors in the training-, validation- and entire group, respectively. AJCC stage and risk score were independent prognostic factors for COAD patients. COAD: colon adenocarcinoma, OS: overall survival, AJCC: American Joint Committee on Cancer.

We identified independent prognostic factors for COAD patients. Variables included age, sex, AJCC stage, T stage, N stage, M stage, and risk scores were included in the analysis. In univariate Cox analysis, there were statistically significant differences among AJCC stage, T stage, N stage, M stage, and risk score (Figure 3A). In the following analysis, AJCC stage was included, but not T stage, N stage, or M stage. In multivariate cox analysis, AJCC stage (hazard ratio [HR]: 1.748; 95% confidence interval [CI]: 1.318-2.318) and risk score (HR: 1.085; 95% CI: 1.049-1.123) were independent prognostic factors for COAD patients (Figure 3B). What’s more, we also confirmed those results in the validation- and the entire group (Figure 3C-3F).

### Correlation between the expression level of seven ferroptosis-related lncRNAs and clinicopathological features

**FIGURE 4:**
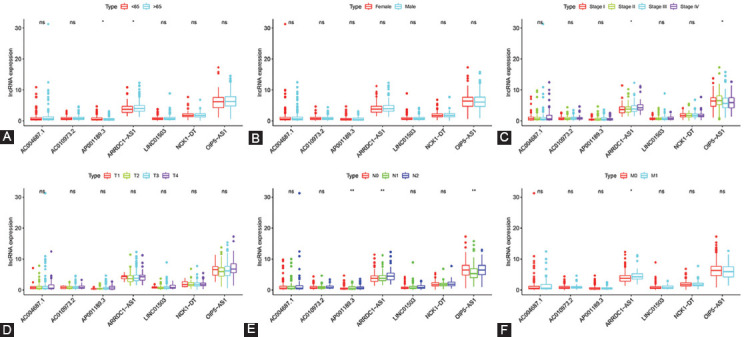
Correlation between the expression level of seven ferroptosis-Related lncRNAs and clinicopathological features. (A-F) Age, sex, AJCC stage, pT stage, pN stage, and pM stage, respectively. AJCC: American Joint Committee on Cancer, NS: not significant. *P < 0.05, **P < 0.01.

Next, we explored the relationship between the expression of each lncRNA and clinicopathological features. In detail, for age or sex, no statistical difference was observed in the expression level of each lncRNA (Figure 4A,4B). For AJCC stage, a higher AJCC stage, a higher expression level of ARRDC1−AS1 and OIP5−AS1. However, this phenomenon was not observed in AC004687.1, AC010973.2, AP001189.3, LINC01503, and NCK1−DT (Figure 4C). The expression level of seven ferroptosis-related lncRNAs was not associated with pT stage (Figure 4D). Furthermore, pN stage showed a statistically significant correlation with the distribution level of AC010973.2, ARRDC1−AS1, and OIP5−AS1 (Figure 4E). Only the expression level of ARRDC1−AS1 was significantly correlated with pM stage (Figure 4F).

### PCA and GSEA

**FIGURE 5:**
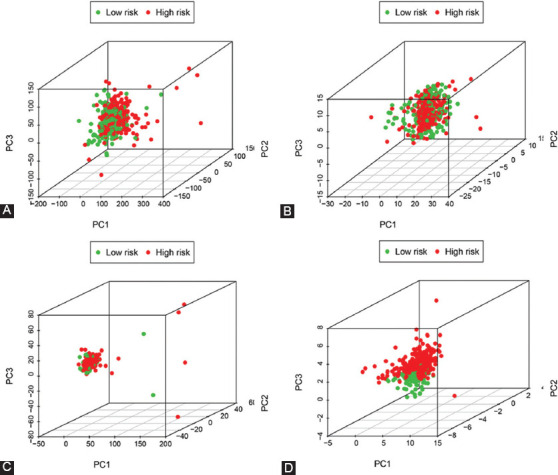
Principal component analysis. PCA plots depicted distinct distribution of high- and low-risk groups based on (A) All genes, (B) ferroptosis-related genes, (C) ferroptosis-related lncRNAs, and (D) ferroptosis-related lncRNAs prognostic signature. Based on ferroptosis-related lncRNAs prognostic signature, COAD patients were divided into two clusters. COAD: colon adenocarcinoma.

**FIGURE 6:**
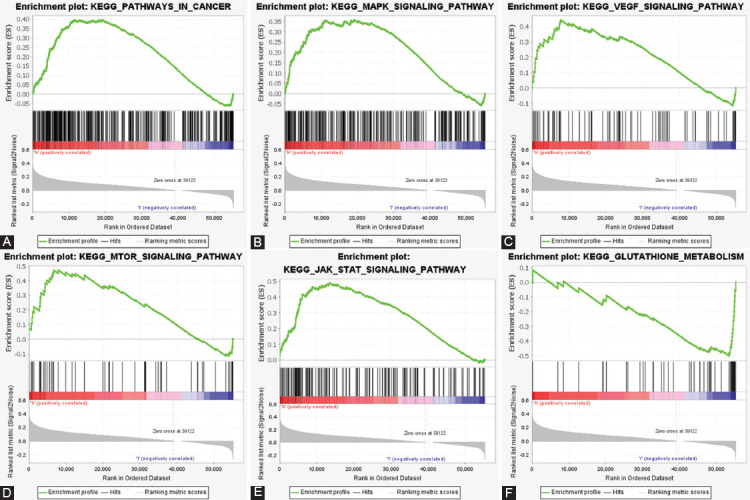
Gene set enrichment analysis. Cancer-related signaling pathways were enriched in the high-risk group, including (A) pathways in cancer, (B) MAPK signaling pathway, (C) VEGF signaling pathway, (D) mTOR signaling pathway, and (E) JAK signaling pathway. (F) Ferroptosis-related signaling pathways were enriched in the high-risk group, including (F) glutathione metabolism. All P < 0.05, FDR<0.25.

To explore the distribution of high- and low-risk groups, we performed PCA (Figure 5). Intuitively, based on the model lncRNAs, we observed COAD patients were divided into two clusters. Next, we utilized GSEA for explored the potential biologic function of seven ferroptosis-related LncRNAs. Results from GSEA presented that several tumor-related pathways were enriched in the high-risk group, such as pathways in cancer, MAPK signaling pathway, VEGF signaling pathway, mTOR signaling pathway, and JAK signaling pathway. Meanwhile, the low-risk group was enriched in ferroptosis-related biological pathways, including glutathione metabolism, as shown in Figure 6.

## DISCUSSION

Recently, although advances in surgery and chemotherapy, the prognosis of patients with advanced and metastatic COAD remained unsatisfactory[16]. Furthermore, patients with the same TNM stage or similar risk factors may have distinct clinical outcomes because of the molecular heterogeneity. Consequently, the role of molecular biomarkers prognosis is of particular importance[17]. Ferroptosis was involved in COAD migration, invasion, proliferation, and other biological behaviour [6, 7]. Available studies have demonstrated that lncRNAs perform a crucial regulatory role in ferroptosis- related biological processes in various cancer types [18]. However, a prognostic tool basing on ferroptosis- related lncRNAs for COAD patients is still lacking.

In the current study, we constructed seven ferroptosis-related lncRNAs signature to predict OS in patients with COAD. Firstly, 259 ferroptosis-related genes and 905 ferroptosis-related lncRNAs were obtained. Patients with COAD were randomly assigned to training- and validation groups. Based on the training group, prognostic ferroptosis-related lncRNAs were confirmed using LASSO regression and Cox regression model. According to the risk score, patients were categorized into a high- and low-risk group. OS between high- and low-risk groups was statistically significant. AUC also suggests the prediction ability of lncRNAs signature. Cox analysis further revealed the risk score was an independent prognostic factor of COAD. High- and low-risk groups were discriminated against intuitively with PCA analysis. Besides, this model was validated in the validation- and the entire group. Lastly, we identified seven lncRNAs signature, including LINC01503, AC004687.1, AC010973.2, AP001189.3, ARRDC1-AS1, OIP5-AS1, and NCK1-DT.

OIP5-AS1 was found to be overexpressed in various tumors, such as lung cancer, breast cancer, cervical cancer, bladder cancer, and other tumor types[19, 20]. In general, high OIP5-AS1 expression was correlated with poorer survival[21]. One study suggested that in radio-resistant colorectal cancer cells, OIP5-AS1 was down expressed[22]. OIP5-AS1overexpression leads to a decrease in cancer cell survival and enhances the sensitivity to radiotherapy. A recent study demonstrated that ferroptosis inducers improved cancer cell radiotherapy resistance[23]. Radiotherapy leads to increased ferroptosis, which improved treatment responses and survival. Therefore, the relationship between OIP5-AS1and ferroptosis should be further investigated. LINC01503 was reported to play a characterized oncogenic role in tumors. Xie et al. revealed that compared with that in adjacent non-tumor tissues, the expression of LINC01503 was significantly higher in esophageal squamous cell carcinoma[24]. Esophageal squamous cell proliferation, migration, and invasion were related to the enhanced LINC01503 expression. Ma et al. showed that LINC01503 was highly expressed in gastric cancer and significantly impact the prognosis of patients with gastric cancer[25]. The involvement of LINC01503 in multiple biological processes has been reported in other tumor types. In several tumor types, including glioma, ARRDC1-AS1 was upregulated, and its expression was associated with the development of clinical progression and survival of patients with glioma[26]. Besides, there are few reports on AC004687.1, AC010973.2, AP001189.3, and NCK1-DT. Thus, our future studies will focus on those lncRNAs.

Afterwards, using the GSEA, we revealed potential signal pathways of seven ferroptosis-related lncRNAs. Pathway in cancer, mTOR signaling pathway, MAPK signaling pathway, VEGF signaling pathway, and JAK-STAT signaling pathway were significantly enriched in the high-risk group (P < 0.05, FDR<0.25). Previous studies have been confirmed that mTOR-, MAPK- and JAK-STAT signaling pathway was involved in the regulation of ferroptosis in diverse diseases[27-29]. Zhang et al. identified that mTOR signaling pathway was activated in COAD, leading to lethal ROS accumulation and ferroptosis[30, 31]. On the other hand, we observed that in the low-risk group, the glutathione metabolism pathway was enriched. Indeed, glutathione participated in antioxidant defences intracellularly, and inhibition of glutathione synthesis is a crucial step of ferroptosis[32, 33]. The results indicated that seven ferroptosis-related lncRNAs exerted significant effects in the ferroptosis process.

In recent years, we have witnessed ferroptosis has played a crucial role in eliminating cancer cells and overcoming therapy resistance[18]. On the other hand, the biological function of lncRNA has gradually become elucidated. Some lncRNA influence cancer progression and therapeutic effect via diverse biological ways[34]. Nevertheless, many unexplored areas remain between ferroptosis and lncRNA. Herein, we utilized high-throughput sequencing data to determine seven ferroptosis-related lncRNAs. Moreover, we look forward to providing a promising target for future investigations.

Inevitably, some limitations of the present study exist. To test the effectiveness of lncRNAs signature, we used only the TCGA validation group and the entire group. More additional patients can improve the reliability of this model. Further experimental studies are required to clarify the biological mechanisms of ferroptosis-related lncRNAs.

## CONCLUSION

In summary, we developed a score model base on seven ferroptosis-related (LINC01503, AC004687.1, AC010973.2, AP001189.3, ARRDC1-AS1, OIP5-AS1, and NCK1-DT). This novel model may provide new research strategies in exploring the mechanisms of ferroptosis and present individualized predictions for CAOD patients’ prognosis.
